# Clinical impact of abdominal versus mediastinal metastases as a prognostic factor for poor outcomes following esophageal cancer surgery: a retrospective study

**DOI:** 10.1186/s12885-021-08484-2

**Published:** 2021-06-23

**Authors:** Yutaka Miyawaki, Hiroshi Sato, Shuichiro Oya, Hirofumi Sugita, Yasumitsu Hirano, Shinichi Sakuramoto, Kojun Okamotom, Shigeki Yamaguchim, Isamu Koyama

**Affiliations:** Department of Gastroenterological Surgery, Saitama Medical University International Medical Center, 1397-1 Yamane, Hidaka-shi, Saitama, 350-1298 Japan

**Keywords:** Abdominal lymph node metastasis, Esophageal cancer, Survival

## Abstract

**Background:**

Surgery is still the mainstay of radical treatment for resectable esophageal cancer (EC). It is apparent that the presence or spread of lymph node metastasis (LNM) is a powerful prognostic factor in patients with EC who are eligible for curative treatment. Although the importance and efficacy of lymph node dissection in radical esophagectomy have been reported, the clinical or prognostic relevance of specific metastatic patterns within the mediastinal cavity and abdomen remains unclear.

**Methods:**

We retrospectively analyzed the association of postoperative survival with clinical mediastinal LNM (cMLNM) and abdominal LNM (cALNM) in 157 patients who underwent radical EC surgery at our hospital between May 2012 and March 2018.

**Results:**

A significant difference in cause-specific survival (CSS) was observed between patients with and without cALNM (log-rank *p* = 0.000). A multivariate Cox regression analysis revealed that cALNM and thoracic surgery (mediastinal lymphadenectomy via conventional open right thoracotomy or video-assisted thoracoscopic surgery) independently predicted CSS (*p* = 0.0007 and 0.021, respectively). Moreover, a significant difference in systemic recurrence-free survival was observed between those with and without cALNM (log-rank *p* = 0.000). Multivariate Cox regression analysis revealed that cALNM and sex independently predicted systemic recurrence-free survival (*p* = 0.000 and 0.015, respectively).

**Conclusion:**

cALNM was an independent poor prognostic factor for CSS after EC surgery. It may also be an independent prognostic factor for postoperative systemic recurrence, which can shorten the CSS. For patients with cALNM-positive EC who have a high potential risk of systemic metastases, more extensive treatment besides the conventional perioperative systemic chemotherapy may be necessary.

## Background

Surgery is the mainstay of radical treatment for resectable esophageal cancer (EC) worldwide [1, 2]. Many studies have been conducted on factors that affect the outcomes following EC surgery, including clinicopathological and surgery-related factors. In particular, the presence of lymph node metastasis (LNM) is a powerful prognostic factor in patients with EC who are eligible for curative treatment.

The extent of LNM associated with EC can anatomically span multiple areas, such as the neck, chest, and abdomen, and the risk of LNM is high even in the early stages [3]. Our previous study on the oncological tolerability of minimally invasive esophagectomy (MIE) suggested that clinical abdominal LNM (cALNM) is a poor prognostic factor after EC surgery [4]. The clinical or prognostic relevance of specific metastatic patterns within the mediastinal cavity and abdomen remain unclear, although the importance and efficacy of lymph node dissection in radical esophagectomy have been reported [5, 6].

Here, we performed a detailed study to assess the significance of clinical mediastinal LNM (cMLNM) and cALNM as postoperative prognostic factors in patients with EC.

## Methods

### Patients

We identified 216 consecutive patients who had undergone radical esophagectomy with two- or three-field lymphadenectomy for thoracic EC at our Department of Gastroenterological Surgery in Saitama Medical University International Medical Center between May 2012 and May 2018. See the details regarding the exclusion criteria below. After applying these criteria, 157 cases were eligible for inclusion. The clinicopathological characteristics and postoperative outcomes were collected from the patients’ records.

### The exclusion criteria

The exclusion criteria were as follows: patients with salvage surgery after definitive radiation therapy, those with obvious residual lesions identified intraoperatively, those who underwent a planned two-stage split surgery, those with clinical distant metastases as defined in the 8th edition of the International Union Against Cancer guidelines [7], and those who underwent esophageal reconstruction using methods other than a gastric tube. In addition, patients with an observation period of less than 180 days were excluded. Moreover, since it is apparent that the number of harvested lymph nodes during surgery affects the prognosis, patients with a small number of harvested lymph nodes (less than 10) were excluded.

### Clinical tumor–node–metastasis staging and follow-up after surgery

Tumor staging was performed according to the 8th edition of the International Union Against Cancer guidelines [7]. Any metastases from the neck to the abdomen were identified using computed tomography (CT) and fluorodeoxyglucose-positron emission tomography, which are routinely performed for the purpose of searching for metastasis before treatment. Follow-up CT assessments every 2–4 months and annual esophagogastroduodenoscopy after surgery are performed for 5 years if no recurrence was suspected.

### Surgical procedure

A transthoracic en bloc resection was performed in all patients with either a two field (mediastinal and abdominal) or three field (mediastinal, abdominal, and cervical) lymph node dissection. In the thoracic part, we performed the conventional open right thoracotomy or video-assisted thoracoscopic surgery (VATS) in the left lateral position while the patients were receiving separate lung ventilation. In the abdominal part, we performed abdominal lymphadenectomy and mobilization of the stomach for the reconstructed organ via conventional open laparotomy or hand-assisted laparoscopic surgery (HALS). The details of the procedure for the abdominal part have been described previously [4]. Overall, the extent of the lymph node dissection was D2 according to the Japanese Classification of Esophageal Cancer (11th Edition) [8]. In particular, the upper, middle, and lower mediastinal lymph nodes dissection was performed including the cervical and thoracic paraesophageal, right and left recurrent nerve, subcarinal, right and left main bronchus, supradiaphragmatic, and anterior thoracic paraaortic lymph nodes. In the abdomen, an upper abdominal and retroperitoneal lymph node dissection was performed including the paracardial, lesser curvature, left gastric, common hepatic, splenic, and celiac lymph nodes.

### Perioperative therapy

Perioperative therapy, including neoadjuvant chemotherapy (NAC) and adjuvant chemotherapy (AC), was administered in accordance with the Japanese Esophageal Society guidelines [1]. Therefore, in principle, NAC was administered to patients who were indicated for radical surgery, except for patients with clinical stage I disease. If radical resection was confirmed pathologically, AC was not administered to patients with squamous cell carcinoma, but only to a small proportion of patients who had adenocarcinoma with multiple positive pathological LNMs. Only six patients received AC in our cohort.

### Postoperative survival and recurrence

The initial recurrence was divided into two types: locoregional and systemic. Locoregional recurrence encompassed resectable mediastinal and abdominal lymph node recurrences, as well as cervical paraesophageal or supraclavicular lymph node recurrence. Systemic recurrence included the following: distant metastases to other organs such as the liver or lungs, unresectable recurrence such as dissemination to the pleura or pericardium, cervical lymph node recurrence other than at the paraesophageal or supraclavicular areas, and recurrence at the para-abdominal aortic lymph nodes. If locoregional and systemic recurrence were observed at the same time, they were treated as systemic recurrence. Locoregional recurrence-free survival (L-RFS) was calculated from the date of the esophagectomy until locoregional recurrence was confirmed, and when any systemic recurrences were confirmed as initial recurrence, they were censored. Likewise, systemic recurrence-free survival (S-RFS) was calculated from the date of the esophagectomy until systemic recurrence was confirmed, and when any locoregional recurrences were confirmed as an initial recurrence, they were censored.

### Statistical analyses

The groups were compared using the Chi-square test or Fisher’s exact test for categorical variables and the Mann-Whitney U test for continuous variables, as appropriate. The Kaplan-Meier method and log-rank test were used to evaluate differences in cause-specific survival (CSS) and recurrence-free survival (RFS). Univariate and multivariate survival analyses were also performed using a stratified Cox proportional hazards model. In the multivariate analysis, covariates were selected by backward elimination. Differences were considered statistically significant at two-tailed *p*-values of < 0.05. All statistical analyses were performed using SPSS software (version 24.0; IBM Corp., Armonk, NY).

## Results

### Clinical mediastinal or abdominal lymph node metastases

Table 1 provides details of the patient characteristics. Of all 157 patients, 70 (44.6%) were clinically positive for LNM, 51 (32.5%) had cMLM, 45 (28.7%) had cALM, 25 (15.9%) were positive for solitary cMLM, 19 (12.1%) were positive for solitary cALM, and 26 (16.6%) were positive for both.

**Table 1:** Differences in clinical factors between patients with and without mediastinal or abdominal lymph node metastases

Factor	Variables	All patients (*N* = 157)	cMLNM^a^			cALNM^b^		
			Absent (*N* = 106)	Present (*N* = 51)	*p* value	Absent (*N* = 112)	Present (*N* = 45)	*p* value
Sex	Male	132	88	44		94	38	
	Female	25	18	7	0.393	18	7	0.573
Age (years)	Mean ± SD^c^	69 ± 7.0	69.2 ± 7.2	68.3 ± 8.8	0.895	69 ± 6	67 ± 8	0.091
Body mass index	Mean ± SD	21.6 ± 2.9	21.7 ± 3.2	21.8 ± 2.7	0.734	21.7 ± 3.0	21.4 ± 2.7	0.604
Tumor location	Ut^d^	16	9	7		15	1	
	Mt^e^	59	38	21		47	12	
	Lt^f^	82	59	23	0.383	50	32	0.006*
Histology	SCC^g^	134	93	41		98	36	
	non-SCC	23	13	10	0.164	14	9	0.17
Neoadjuvant chemotherapy	Absent	80	65	15		67	13	
	Present	77	41	36	0.000*	45	32	0.000*
Adjuvant chemotherapy	Absent	150	103	47		110	40	
	Present	7	3	4	0.155	2	5	0.021*
Clinical tumor depth	T1	58	50	8		53	5	
	T2	21	15	6		14	7	
	T3	98	41	37	0.000*	45	33	0.000*
Clinical mediastinal lymph node metastasis	Absent	106	106	0		87	19	
	Present	51	0	51	0.000*	25	26	0.000*
Clinical abdominal lymph node metastasis	Absent	112	87	25		112	0	
	Present	47	19	26	0.000*	0	47	0.000*
Thoracic approach	OT^h^	63	31	32		34	29	
	VATS^i^	94	75	19	0.000*	78	16	0.000*
Abdominal approach	OL^j^	65	38	26		36	28	
	HALS^k^	77	68	25	0.052	76	17	0.001*

^a^*cMLNM* clinical mediastinal lymph node metastasis, ^b^*cALNM* clinical abdominal lymph node metastasis, ^c^*SD* standard deviation, ^d^*Ut* upper thoracic, ^e^*Mt* middle thoracic, ^f^*Lt* lower thoracic, ^g^*SCC* squamous cell carcinoma, ^h^*OT* open thoracotomy, ^I^*VATS* video-assisted thoracoscopic surgery, ^j^*OL* open laparotomy, ^k^*HALS* hand-assisted surgery. *p < 0.05

### Associations between clinical mediastinal or abdominal lymph node metastases and clinicopathological factors

As shown in Table 1, the cMLNM-positive group included many patients with NAC, deeper clinical tumor invasion depth, cALNM, and VATS because many of them were at an advanced stage. The same was observed in the cALNM-positive group. In addition, many patients with HALS were included in the cALNM group. The frequency of cALNM was low in patients with upper thoracic EC. Moreover, many patients who underwent AC were included in the cALNM-positive group due to the indication of AC, described above.

Likewise, as shown in Table 2, both the cMLNM-positive group consisted of patients with deeper pathological tumor depth invasion and both mediastinal and abdominal pathological LNM. The same was observed in the cALNM-positive group. The frequency of vascular invasion was high in the cMLNM-positive group, while lymphatic invasion was high in the cALNM-positive group. The mean and median number of the harvested lymph nodes was 30.9 (standard deviation, 13.1) and 30.0 (range, 10–99), respectively. The association between the number of harvested lymph nodes and the presence of cMLNM or cALNM was not significant. However, the cMLNM-positive group had more harvested lymph nodes in the cervix whereas the cALNM-positive group had more harvested lymph nodes in the abdomen. More pathological LNM in the cervix, mediastinum, and abdomen were confirmed in the cMLNM-positive group. On the other hand, the cALNM-positive group had more pathological LNM in the mediastinum and abdomen. Intraoperative bleeding was high in both the cMLNM- and cALNM-positive groups, although there were no significant differences between the groups in terms of the total operative time and the length of postoperative hospital stay. Although patients with clinical distant metastases were excluded as described above, none of the patients had pathological distant metastases including supraclavicular LNM.

**Table 2:** Pathological and surgery-related factors in patients with and without clinical mediastinal/abdominal lymph node metastases

Factor	Variables	All patients (*N* = 157)	cMLNM^a^			cALNM^b^		
			Absent (*N* = 106)	Present (*N* = 51)	*p* value	Absent (*N* = 112)	Present (*N* = 45)	*p* value
Pathological T factor	pT1	79	63	15		68	10	
	pT2	21	10	11		12	9	
	pT3	55	31	24		31	24	
	pT4a	3	2	1	0.004*	1	2	0.000*
Pathological N factor	pN0	77	65	12		70	7	
	pN1	49	25	24		30	19	
	pN2	25	12	13		11	14	
	pN3	6	4	2	0.000*	1	5	0.000*
Lymphatic invasion	Absent	99	70	29		79	20	
	Present	58	36	22	0.292	33	25	0.003*
Vascular invasion	Absent	79	61	18		60	19	
	Present	78	45	33	0.011*	52	26	0.22
Intramural metastasis	Absent	131	100	44		106	38	
	Present	11	6	7	0.120	6	7	0.052
Number of harvested lymph nodes	Mean ± SD^c^	30.9 ± 13.1	29.7 ± 13.2	33.3 ± 12.6	0.051	30.0 ± 13.6	33.0 ± 11.5	0.071
	Median (range)	30.0 (10–99)	28.0 (11–99)	33.0 (10–63)		28.5 (11–99)	32.0 (10–58)	
Number of harvested cervical lymph nodes	Mean ± SD	2.2 ± 5.2	1.4 ± 4.4	3.8 ± 6.3	0.002*	2.2 ± 5.5	2.1 ± 4.6	0.362
Number of harvested mediastinal lymph nodes	Mean ± SD	14.6 ± 8.0	14.7 ± 8.6	14.5 ± 6.8	0.856	14.9 ± 8.5	14.0 ± 6.7	0.867
Number of harvested abdominal lymph nodes	Mean ± SD	14.1 ± 7.1	13.6 ± 6.6	15.0 ± 8.8	0.567	13.0 ± 6.4	16.7 ± 8.8	0.019*
Number of lymph node metastases	Mean ± SD	2.2 ± 6.9	1.8 ± 4.2	3.0 ± 6.1	0.000*	1.3 ± 6.5	4.5 ± 7.1	0.000*
	Median (range)	0.0 (0–69)	0.0 (0–69)	0.5 (0–39)		0.0 (0–69)	2.0 (0–39)	
Number of cervical lymph node metastases	Mean ± SD	0.1 ± 0.3	0.0 ± 0.2	0.1 ± 0.4	0.007*	0.0 ± 0.3	0.1 ± 0.4	0.247
Number of mediastinal lymph node metastases	Mean ± SD	0.8 ± 3.8	0.7 ± 4.4	1.1 ± 1.9	0.000*	0.7 ± 4.3	1.2 ± 2.1	0.000*
Number of abdominal lymph node metastases	Mean ± SD	1.3 ± 3.9	1.1 ± 3.2	1.8 ± 4.9	0.011*	0.5 ± 2.4	3.2 ± 5.8	0.000*
Total operative time (min)	Mean ± SD	429.5 ± 69.8	427.1 ± 69.6	434.5 ± 74.9	0.453	432.8 ± 69.7	431.5 ± 75.0	0.308
	Median (range)	435.0 (242–571)	429.0 (248–571)	440.0 (242–562)		437.5 (291–563)	431.0 (242–571)	
Total intraoperative bleeding (ml)	Mean ± SD	247.9 ± 223.7	219.4 ± 198.7	307.0 ± 258.4	0.014*	210.8 ± 142.7	360.0 ± 339.2	0.039*
	Median (range)	174.0 (40–1553)	160.0 (40–1553)	269.0 (50–1511)		161.5 (42–830)	261.0 (40–1553)	
Length of postoperative hospital stay (days)	Median (range)	15 (8–239)	15 (8–106)	19 (9–239)	0.072	15 (8–239)	18 (9–58)	0.484

^a^*cMLNM* clinical mediastinal lymph node metastasis, ^b^*cALNM* clinical abdominal lymph node metastasis, ^c^*SD* standard deviation

### Associations among clinical, surgery-related factors, and postoperative cause-specific survival

**Fig. 1:**
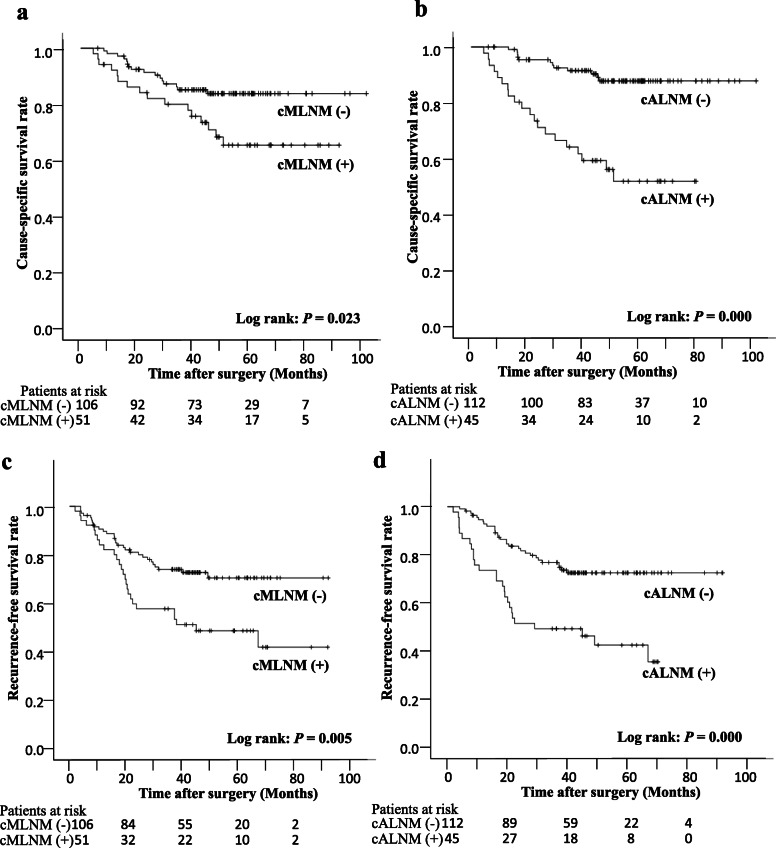
Kaplan-Meier curves for CSS and RFS in all the patients. **a** A significant difference in CSS was observed between patients with and without cMLNM (3-year survival rates: 84.9 and 79.7%, respectively; *p* = 0.023). **b** A significant difference in CSS was observed between those with and without cALNM (3-year survival rates: 91.4 and 63.7%, respectively; *p* = 0.000). **c** A significant difference in RFS was observed between those with and without cMLNM (3-year survival rates: 73.7 and 57.3%, respectively; *p* = 0.005). **d** A significant difference in RFS was also observed between those with and without cALNM (3-year survival rates: 76.6 and 48.9%, respectively; *p* = 0.000). CSS: cause-specific survival, cMLNM: clinical mediastinal lymph node metastasis, cALNM: clinical abdominal lymph node metastasis, RFS: recurrence-free survival

The median postoperative follow-up period was 48.5 (range; 4.4–10.4) months, and 32 cause-specific EC deaths occurred during the entire observational period. A significant difference in CSS was observed between patients with and without cMLNM and cALNM (*p* = 0.023 and 0.000, respectively) (Fig. 1a, b). Additionally, in the univariate analysis, the prognosis was predicted by the absence of AC and different thoracic approaches (*p* = 0.007 and 0.000, respectively). The multivariate Cox regression analysis revealed that the presence of cALNM (*p* = 0.000, hazard ratio = 3.917) and VATS as a thoracic approach (*p* = 0.017, hazard ratio = 0.395) were independent prognostic factors of CSS (Table 3).

**Table 3:** Univariate and multivariate analyses of clinical, surgery-related prognostic factors for postoperative survival

Factor	Category	Cause-specific survival				Recurrence-free survival			
		Univariate	Multivariate			Univariate	Multivariate		
		*p* value	*p* value	HR^a^	(95% CI^b^)	*p* value	*p* value	HR	(95% CI^b^)
Age	< 70 (vs. ≥70)	0.853				0.129			
Sex	Male (vs. Female)	0.796				0.398			
Body mass index	< 20 (vs. ≥20)	0.378				0.328			
Tumor location	Ut^c^ (vs. Mt^d^ or Lt^e^)	0.868				0.791			
Histology	non-SCC^f^ (vs. SCC)	0.113				0.017*	0.088	1.760	(0.919–3.369)
Neoadjuvant chemotherapy	Present (vs. Absent)	0.689				0.138			
Adjuvant chemotherapy	Present (vs. Absent)	0.007*	0.325	1.731	(0.581–5.155)	0.120			
cT factor	cT2–3 (vs. T1)	0.054	0.871	0.926	(0.366–2.343)	0.004*	0.205	1.558	(0.784–3.094)
Clinical mediastinal lymph node metastasis	Present (vs. Absent)	0.023*	0.811	0.908	(0.413–1.998)	0.005*	0.699	1.131	(0.607–2.108)
Clinical abdominal lymph node metastasis	Present (vs. Absent)	0.000*	0.000*	3.917	(1.860–8.250)	0.000*	0.007*	2.162	(1.237–3.781)
Thoracic approach	VATS^g^ (vs. OT^h^)	0.000*	0.017*	0.387	(0.178–0.842)	0.001*	0.021*	0.514	(0.292–0.902)
Abdominal approach	HALS^i^ (vs. OL^j^)	0.170				0.100			
Reconstruction route	mediastinal or antethoracic (vs. Retrosternal)	0.105				0.398			

^a^*HR* hazard ratio, ^b^*CI* confidence interval, ^c^*Ut* Upper thoracic, ^d^*Mt* Middle thoracic, ^e^Lt Lower thoracic, ^f^*SCC* squamous cell carcinoma, ^g^*VATS* Video assisted thoracic surgery, ^h^*OT* Open thoracotomy, ^i^*HALS* Hand assisted laparoscopic surgery, ^j^*OL* Open Laparotomy. *: *p* < 0.05

### Associations among clinical factors, surgery-related factors, and postoperative recurrence-free survival

During the entire observational period, 55 patients had postoperative recurrence. A significant difference in RFS between those with and without cMLNM and cALNM was observed (*p* = 0.005 and 0.000, respectively) (Fig. 1c, d). Additionally, in the univariate analysis, the prognosis was predicted by the difference in histology, deeper tumor depth invasion, and different thoracic approaches (*p* = 0.017, 0.004, and 0.001, respectively). The multivariate Cox regression analysis revealed that the presence of cALNM (*p* = 0.007, hazard ratio = 2.162) and VATS as a thoracic approach (*p* = 0.021, hazard ratio = 0.514) were independent prognostic factors of RFS (Table 3).

### Associations among clinical factors, surgery-related factors, and locoregional or systemic recurrence-free survival

To verify the risk assessment by the recurrence pattern, we classified the initial postoperative recurrence into two types: locoregional or systemic. Overall, 30 and 25 patients had locoregional and systemic recurrence, respectively, during the entire observational period. Table 4 shows the initial postoperative recurrence sites in patients with or without cMLNM or cALNM.

**Table 4:** Initial postoperative recurrence site details in patients with or without clinical mediastinal/abdominal lymph node metastases

Initial recurrence pattern	Variables		All patients (*N* = 157)	(Recurrence site^a^)	cMLNM^b^				cALNM^c^			
					Absent (*N* = 106)		Present (*N* = 51)		Absent (*N* = 112)		Present (*N* = 45)	
Locoregional recurrence	Absent		127		90		37		93		34	
	Present	Cervical lymph node	30	(11)	16	(6)	14	(5)	19	(5)	11	(6)
		Mediastinal local or lymph node		(25)		(12)		(13)		(13)		(12)
		Abdominal lymph node		(10)		(7)		(3)		(5)		(5)
Systemic recurrence	Absent		132		93		39		102		30	
	Present	Lung	25	(9)	13	(4)	12	(5)	10	(3)	15	(6)
		Liver		(6)		(3)		(3)		(2)		(4)
		Para-abdominal aortic lymph node		(6)		(5)		(1)		(2)		(4)
		Pleura		(9)		(3)		(6)		(4)		(5)
		Others (Adrenal gland, bone, or ventricle)		(4)		(2)		(2)		(1)		(3)

^a^Including duplications, ^b^*cMLNM* clinical mediastinal lymph node metastasis, ^c^*cALNM* clinical abdominal lymph node metastasis

**Fig. 2:**
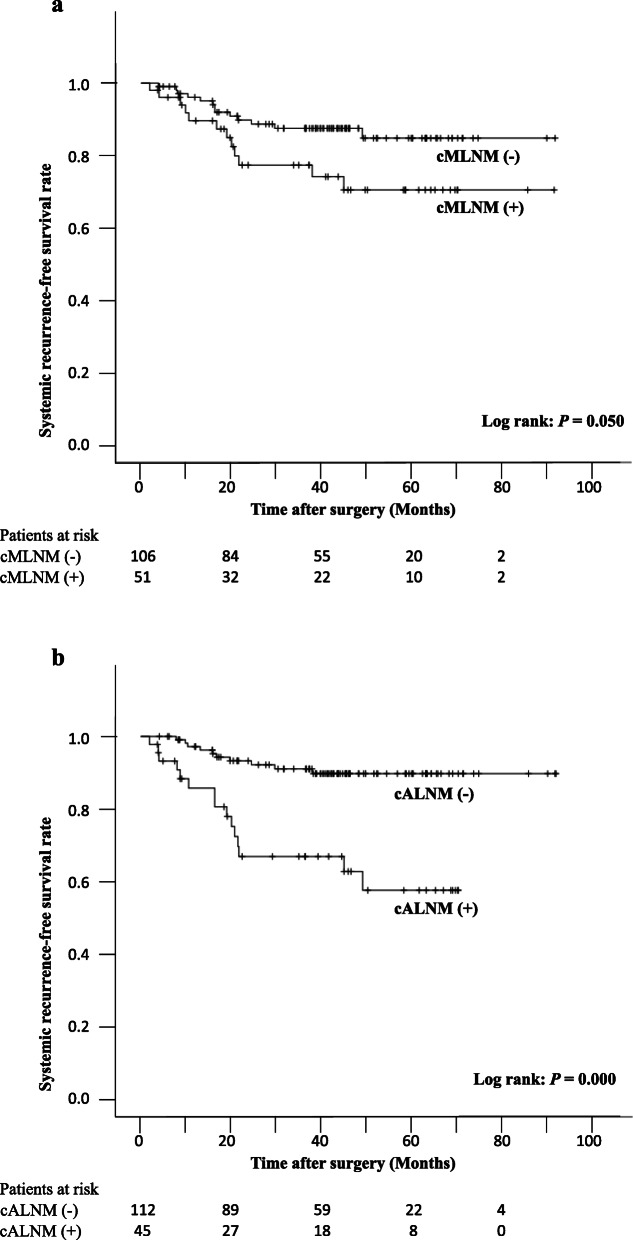
Kaplan-Meier curves for S-RFS in all the patients. **a** There was no statistically significant difference between those with and without cMLNM in terms of S-RFS (*p* = 0.050). **b** A significant difference in S-RFS was observed between those with and without cALNM (3-year survival rates: 89.7 and 66.7%, respectively; *p* = 0.000). cMLNM: clinical mediastinal lymph node metastasis, S-RFS: systemic recurrence-free survival, cALNM: clinical abdominal lymph node metastasis

Although there was no statistically significant difference between those with and without cMLNM for S-RFS (*p* = 0.050), a significant difference in S-RFS was observed between those with and without cALNM (*p* = 0.000) (Fig. 2a, b). Additionally, the univariate analysis revealed that the prognosis was predicted by differences in sex, absence of AC, and different thoracic approaches (*p* = 0.009, 0.019, and 0.013, respectively). The multivariate Cox regression analysis revealed that the presence of cALNM (*p* = 0.000, hazard ratio = 4.520) and difference in sex (*p* = 0.015, hazard ratio = 2.759) independently predicted S-RFS. (Table 5). On the other hand, significant differences in L-RFS were observed between those with and without cMLNM, NAC, clinical tumor depth invasion, and thoracic approach (*p* = 0.045, 0.040, 0.021, and 0.018, respectively). However, the multivariate Cox regression analysis revealed that only deeper clinical tumor depth invasion independently predicted L-RFS (*p* = 0.027, hazard ratio = 2.743). (Table 5).

**Table 5:** Univariate and multivariate analyses of prognostic factors for locoregional and systemic relapse-free survival

Factor	Category	Locoregional recurrence-free survival				Systemic recurrence-free survival			
		Univariate	Multivariate			Univariate	Multivariate		
		*p* value	*p* value	HR^a^	(95% CI^b^)	*p* value	*p* value	HR	(95% CI)
Age	< 70 (vs. ≥70)	0.057				0.408			
Sex	Male (vs. Female)	0.707				0.009*	0.015*	2.759	(1.217–6.253)
Body mass index	< 20 (vs. ≥20)	0.425				0.566			
Tumor location	Ut^c^ (vs. Mt^d^ or Lt^e^)	0.224				0.338			
Histology	non-SCC^f^ (vs. SCC)	0.368				0.292			
Neoadjuvant chemotherapy	Present (vs. Absent)	0.040*	0.417	1.486	(0.572–3.862)	0.663			
Adjuvant chemotherapy	Present (vs. Absent)	0.971				0.019*	0.128	2.764	(0.746–10.239)
cT factor	cT2–3 (vs. T1)	0.021*	0.027*	2.743	(1.121–6.714)	0.089			
Clinical mediastinal lymph node metastasis	Present (vs. Absent)	0.045*	0.493	1.318	(0.599–2.897)	0.050	0.898	1.064	(0.411–2.756)
Clinical abdominal lymph node metastasis	Present (vs. Absent)	0.116				0.000*	0.000*	4.520	(2.028–10.072)
Thoracic approach	VATS^g^ (vs. OT^h^)	0.018*	0.104	0.534	(0.250–1.138)	0.013*	0.194	0.569	(0.243–1.332)
Abdominal approach	HALS^i^ (vs. OL^j^)	0.085				0.579			
Reconstruction route	mediastinal or antethoracic (vs. Retrosternal)	0.718				0.104			

^a^*HR* hazard ratio, ^b^*CI* confidence interval, ^c^*Ut* Upper thoracic, ^d^*Mt* Middle thoracic, ^e^*Lt* Lower thoracic, ^f^*SCC* squamous cell carcinoma, ^g^*VATS* Video assisted thoracic surgery, ^h^*OT* Open thoracotomy, ^i^*HALS* Hand assisted laparoscopic surgery, ^j^*OL* Open Laparotomy. *: *p* < 0.05

### Difference in postoperative survival with and without lymph node metastasis in the mediastinal or abdominal fields

**Fig. 3:**
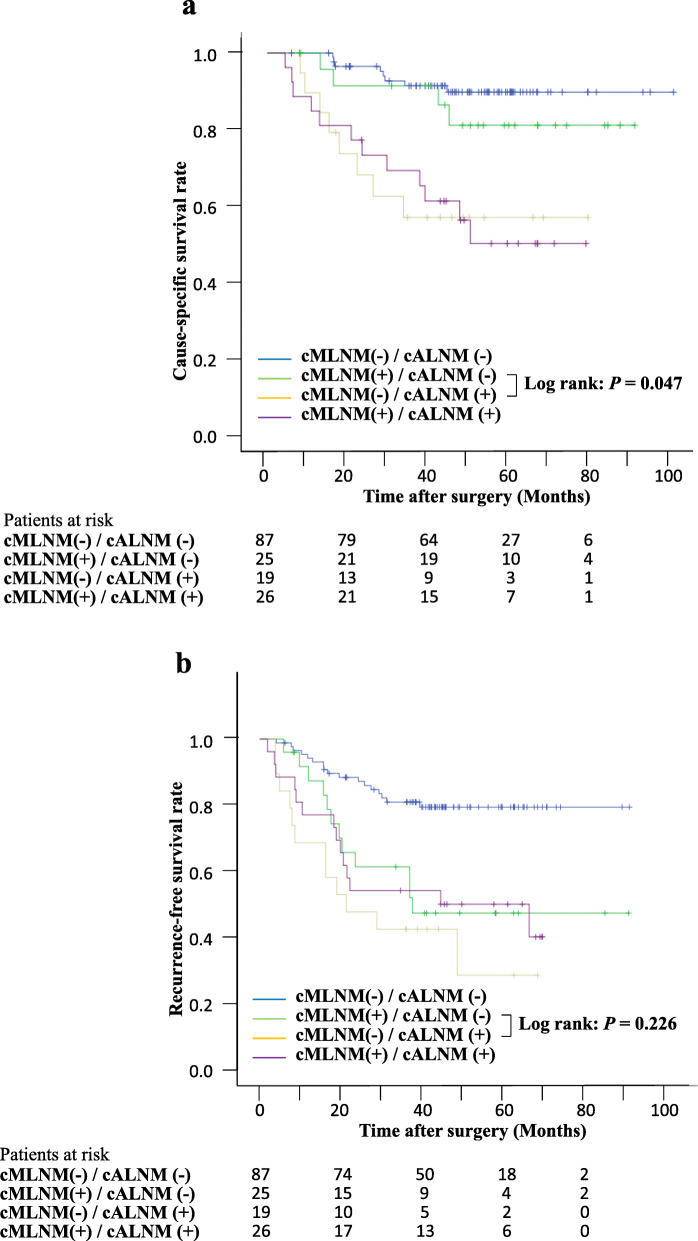
Kaplan-Meier curves for CSS in patients in four groups according to the status of cMLNM and cALNM**. a** A significant difference was observed for CSS among the four (cMLNM[−/+]/cALNM[−/+]) groups (*p* = 0.000), as well as between those who were cMLNM(+)/cALNM(−) and those who were cMLNM(−)/cALNM(+) (3-year survival rates: 91.3 and 56.4%, respectively; *p* = 0.047). **b** A significant difference in RFS was likewise observed among these four groups (*p* = 0.000). There was no statistically significant difference between those who were cMLNM(+)/cALNM(−) and those who were cMLNM(−)/cALNM(+) (*p* = 0.226). CSS: cause-specific survival, cMLNM: clinical mediastinal lymph node metastasis, cALNM: clinical abdominal lymph node metastasis, RFS: recurrence-free survival

To eliminate the bias of including cLNM-negative patients who had a favorable prognosis or both cMLNM- and cALNM-postive patients who had worse prognosis in our study population, we performed a subgroup analysis based on the presence or absence of LNM in the mediastinum and abdomen. We classified patients into four groups: cMLNM(−)/cALNM(−), cMLNM(+)/cALNM(−), cMLNM(−)/cALNM(+), and cMLNM(+)/cALNM(+). The Kaplan-Meier analysis revealed significant differences in CSS and RFS among these four groups (*p* = 0.000 and 0.000, respectively) (Fig. 3a, b). Furthermore, in a subgroup analysis of 44 cases with clinical LNM in only one field (the solitary cMLNM- and solitary cALNM- positive groups), survival rate in CSS was worse in the solitary cALNM-positive group than in the solitary cMLNM-positive group (*p* = 0.047) (Fig. 3a). For RFS, there was no statistically significant difference between those two groups (*p* = 0.226) (Fig. 3b).

### Correlation between the clinical and pathological diagnosis

To verify the correlation between clinical and pathological diagnosis of T and N factors, T factor was divided into two groups, shallower or deeper than T1, and N factor was divided into two groups, presence or absence of LNM. Furthermore, considering the modification of NAC by the antitumor effect, subgroup analysis with and without NAC was also performed. As shown in Table 6, the accuracy rate of T factor was lower in patients who underwent NAC than in all the patients overall and in patients who did not undergo NAC (74.0, 82.2, and 90.0%, respectively). A similar tendency was observed for factor N, but the accuracy rate in each patient group was lower than that for the T factor group (71.4, 73.2, and 75.0%, respectively).

**Table 6:** Correlation between the clinical and pathological diagnoses

Factors	All patients (*n* = 157)					Factors	Patients who did not undergo NAC^a^ (*n* = 80)					Factors	Patients who underwent NAC (n77)				
	pT0–1	pT2-4a	ρ^b^ value	positive predictive value	accuracy rate		pT0–1	pT2-4a	ρ value	positive predictive value	accuracy rate		pT0–1	pT2-4a	ρ value	positive predictive value	accuracy rate
cT1	54	4				cT1	50	4				cT1	4	0			
cT2–3	24	75	0.665	75.8	82.2	cT2–3	4	22	0.772	84.6	90.0	cT2–3	20	53	0.348	72.6	74.0
Factors	All patients (*n* = 157)					Factors	Patients who did not undergo NAC^a^ (*n* = 80)					Factors	Patients who underwent NAC (n77)				
	pN0	pN1–3	ρ value	positive predictive value	accuracy rate		pN0	pN1–3	ρ value	positive predictive value	accuracy rate		pN0	pN1–3	ρ value	positive predictive value	accuracy rate
pN0	61	26				pN0	43	16				pN0	18	10			
pN1–3	16	54	0.470	77.1	73.2	pN1–3	4	17	0.481	81.0	75.0	pN1–3	12	37	0.393	75.5	71.4

^a^*NAC* neoadjuvant chemotherapy, ^b^*ρ* Spearman’s correlation coefficients, *: *p* < 0.05

### Associations among clinical, pathological N factors, and postoperative survival

Since cALNM was suggested as a prognostic clinical and surgical factor, additional risk analysis including pathological factors, which are promising prognostic factors, was performed. As shown in Table 7, the multivariate Cox regression analysis revealed that the presence of cALNM (*p* = 0.030, hazard ratio = 2.437) and pathological ALNM (pALNM) (*p* = 0.001, hazard ratio = 4.611) independently predicted CSS. However, the multivariate Cox regression analysis revealed that only pALNM independently predicted RFS (*p* = 0.000, hazard ratio = 5.111).

**Table 7:** Univariate and multivariate analyses of clinicopathological prognostic factors for postoperative survival

Factor	Category	Cause-specific survival				Recurrence-free survival			
		Univariate	Multivariate			Univariate	Multivariate		
		*p* value	*p* value	HR^a^	(95% CI^b^)	*p* value	*p* value	HR	(95% CI^b^)
Clinical mediastinal lymph node metastasis	Present (vs. Absent)	0.023*	0.654	1.184	(0.565–2.484)	0.005*	0.250	1.381	(0.797–2.392)
Clinical abdominal lymph node metastasis	Present (vs. Absent)	0.000*	0.030*	2.437	(1.092–5.440)	0.000*	0.677	0.873	(0.461–1.654)
Pathological abdominal lymph node metastasis	Present (vs. Absent)	0.000*	0.001*	4.611	(1.800–11.811)	0.000*	0.000*	5.111	(2.878–9.078)

^a^*HR* hazard ratio, ^b^*CI* confidence interval, *: *p* < 0.05

## Discussion

Clinical LNM is a known poor prognostic factor in patients with EC, and the significance of the lymph node dissection for postoperative survival after EC surgery has been verified [9]. The effect of dissection on postoperative survival at each lymph node station has also been verified using an efficacy index [10]. However, the significance of metastasis to the mediastinal or abdominal lymph nodes, which are common sites of metastasis in EC, for postoperative survival, has not been verified yet. Our study on the postoperative long-term prognosis of clinical LNM in the mediastinal and abdominal fields demonstrated that the presence of cALNM was an independent poor prognostic factor for CSS and RFS following EC surgery. Additionally, the presence of cALNM was an independent poor prognostic factor for postoperative systemic recurrence, although neither cMLNM nor cALNM was found to be associated with locoregional recurrence.

In addition, among patients with metastasis in the mediastinum or abdomen alone, those with solitary cALNM had a poorer prognosis than those with solitary cMLNM. These results suggest that cALNM may be a poorer prognostic factor for postoperative survival, even when compared with cMLNM, due to its association with potential systemic recurrence.

Recurrence after radical surgery for EC is, unfortunately, a major clinical issue [11–13]. There are many reports showing the usefulness of radiotherapy or chemoradiotherapy for localized recurrence after EC surgery, and these are widely used in clinical practice [14]. The usefulness of resecting the lymph nodes, including the cervical lymph nodes, for localized postoperative recurrence has also been reported in a retrospective observational study [15]. Most of the treatments for recurrence affecting the organs are not radical treatments; resection has been limited to a small number of patients [16, 17], and its usefulness is unknown [18, 19]. If cALNM is a risk factor for postoperative systemic recurrence, this may be one reason why it was a poor prognostic factor for CSS in our study. Comparison of cLNM in the mediastinal and abdominal fields alone showed a similar survival curve for RFS; however, for CSS, the survival rate of the cALNM group tended to be lower. This appears to support the above hypothesis. Moreover, if cALNM reflects the potential risk of systemic recurrence in patients with EC after radical surgery, the introduction of a more powerful perioperative adjuvant therapy should be considered as systemic treatment for patients with cALNM.

Patients with cALNM who were examined for the postoperative long-term prognosis included both cMLNM-positive and cMLNM-negative patients. It is possible that this had some effect on the assessment of biological malignancy for cALNM. This also applies to patients with cMLNM. However, the proportions of patients who were positive for both cMLNM and cALNM among patients with cMLNM and cALNM were non-significantly different at 26 of 51 (51.0%) vs. 26 of 45 (57.8%), respectively. Moreover, in the subgroup analysis of the presence or absence of LNM in the mediastinal or abdominal field alone, patients with solitary cALNM had significantly shorter CSS than patients with solitary cMLNM (Fig. 3). In addition, the Kaplan-Meier curve in CSS was similar for patients with solitary cALNM and those who were both cMLNM- and cALNM-positive; furthermore, in RFS, the Kaplan-Meier curves of patients with solitary cMLNM, cALNM, and both cMLNM- and cALNM-positive were similar. This result is consistent with the results of the main analysis, and we believe that the effect of this bias was small. Since there are many patients with synchronous cMLNM and cALNM in clinical practice, we thought that it has a certain meaning to assess the biological malignancy of those with cALNM and cMLNM, including those with overlap.

Interestingly, the number of harvested abdominal lymph nodes was significantly higher in cALNM-positive patients than in cALNM-negative patients. Although the extent of lymphadenectomy was the same regardless of the cALNM status, it is speculated that this is partly due to the surgeon trying to perform more aggressive abdominal lymphadenectomy in cALNM-positive cases. As shown in Table 4, only 10 cases (6.4%) were found to have recurrence limited to the abdominal field, and locoregional control was performed to some extent. The finding that cALNM-positive patients had a poor prognosis despite some locoregional control secured by aggressive lymphadenectomy may support our hypothesis that cALNM poses a risk for systemic recurrence.

Even with recent advances in diagnostic modalities, the clinical diagnosis of LNM remains difficult. The presence of numerous micrometastases at the early clinical stages may explain the discrepancies between pathological LNM assessments [20]. Likewise, the effect of preoperative therapy on advanced cancer also contributes to the discrepancy between clinical and pathological stages. In our study, the discrepancy between clinical and pathological diagnoses in patients who underwent NAC was clear from the correlation coefficients, positive predictive value, and accuracy rates compared with those of patients who did not undergo NAC (Table 6). Pathological ALNM was very promising as a prognostic factor in clinicopathological factors (Table 7). On the other hand, it was suggested that cALNM is also an independent prognostic factor of CSS. We considered that cALNM was not the same as pALNM, since cALNM is a prognostic factor that had not been modified by NAC, and it is considered a useful prognostic factor before treatment despite the inaccuracy of the preoperative diagnosis. If a discrepancy between clinical and pathological stages is unavoidable, cALNM, which may reflect the potential risk of postoperative systemic recurrence, may be a different prognostic factor than pALNM.

In our study, likewise, it is necessary to fully consider the accuracy of cALNM diagnosis. Because our study suggests that cALNM reflects a potential risk for postoperative systemic recurrence before cancer treatment, cALNM is considered an important clinical finding that can complement the inaccuracies in preoperative metastasis diagnosis.

In our study, the different thoracic approaches were also demonstrated as independent factors associated with CSS and RFS after EC surgery. Although it remains unclear whether MIE contributes to improved long-term prognosis in those who undergo EC surgery [21], the superiority of its long-term prognosis has been reported in some retrospective studies, which is similar to our results [22]. The patients’ sex was also demonstrated as an independent factor associated with postoperative systemic recurrence, consistent with some reports that identified sex as a prognostic factor in patients undergoing EC surgery [23].

Because cALNM associated with upper thoracic EC has a poor prognosis, it is characterized by group-3 lymph nodes in such patients [8]. It is necessary to consider the difference in the effect of cALNM on prognosis, depending on the tumor location. Because the proportion of patients with upper thoracic EC in our cohort was low at 10.2%, and only 2.2% of those with upper EC and a poor prognosis in the cALNM-positive group were included, we believe that the impact of this on the results was very small.

One major limitation of the current research is that it was a single-institution retrospective study. Therefore, the sample volume was relatively small, so a limitation exists about the conclusion that could be reached. While it is clear that findings from a larger sample size are more convincing, we believe that we have been able to reliably verify the significance of cALNM as a prognostic clinical factor for CSS. Another limitation is that our treatment policy was based on the standard treatment in Japan, which is different from the treatment systems in many foreign countries. In Japan, for advanced cancer, subsequent radical surgery after NAC is considered the standard treatment based on the JCOG9907 trial [1, 24]. On the other hand, in Western countries, the current gold standard of perioperative treatment is neoadjuvant chemoradiotherapy according to the CROSS trial, or perioperative chemotherapy according to the Magic trial [25, 26]. Although re-examining may be necessary in clinical practice with different treatment policies, we believe that our results offer universal prognostic factors for predicting poor prognosis in patients with EC.

## Conclusion

In conclusion, cALNM is an independent poor prognostic factor for CSS and RFS after EC surgery. It may also be an independent prognostic factor for postoperative systemic recurrence, which in turn, may be a mechanism that shortens CSS. For patients with cALNM-positive EC who have a high potential risk of systemic metastasis, a regimen stronger than the conventional perioperative systemic chemotherapy may be necessary to improve the prognosis.

## Data Availability

The datasets used or analyzed during the current study are available from the corresponding author on reasonable request.

## Abbreviations

AC: Adjuvant chemotherapy
cALNM: Clinical abdominal lymph node metastasis
cMLNM: Clinical mediastinal LNM
CSS: Cause-specific survival
CT: Computed tomography
EC: Esophageal cancer
HALS: Hand-assisted laparoscopic surgery
LNM: Lymph node metastasis
L-RFS: Locoregional recurrence-free survival
MIE: Minimally invasive esophagectomy
NAC: Neoadjuvant chemotherapy
RFS: Recurrence-free survival
S-RFS: Systemic recurrence-free survival
VATS: Video-assisted thoracoscopic surgery

## References

[1] Kitagawa Y, Uno T, Oyama T, Kato K, Kato H, Kawakubo H, Kawamura O, Kusano M, Kuwano H, Takeuchi H, Toh Y, Doki Y, Naomoto Y, Nemoto K, Booka E, Matsubara H, Miyazaki T, Muto M, Yanagisawa A, Yoshida M (2019). Esophageal cancer practice guidelines 2017 edited by the Japan esophageal society: part 1. Esophagus..

[2] Lagergren J, Smyth E, Cunningham D, Lagergren P (2017). Oesophageal cancer. Lancet..

[3] Akutsu Y, Kato K, Igaki H, Ito Y, Nozaki I, Daiko H, Yano M, Udagawa H, Nakagawa S, Takagi M, Mizusawa J, Kitagawa Y (2016). The prevalence of overall and initial lymph node metastases in clinical T1N0 thoracic esophageal cancer: from the results of JCOG0502, a prospective multicenter study. Ann Surg.

[4] Miyawaki Y, Sato H, Fujiwara N, Aoyama J, Oya S, Sugita H, Hirano Y, Sakuramoto S, Okamoto K, Yamaguchi S, Koyama I (2021). Verification of oncological local control for hand-assisted laparoscopic abdominal lymph node dissection in esophageal cancer surgery: a propensity score-matched analysis. Esophagus..

[5] Altorki NK, Zhou XK, Stiles B, Port JL, Paul S, Lee PC, Mazumdar M (2008). Total number of resected lymph nodes predicts survival in esophageal cancer. Ann Surg.

[6] Mantziari S, Allemann P, Winiker M, Sempoux C, Demartines N, Schäfer M (2017). Sterilization of tumor-positive lymph nodes of esophageal cancer by neo-adjuvant treatment is associated with worse survival compared to tumor-negative lymph nodes treated with surgery first. J Surg Oncol.

[7] Rice TW, Gress DM, Patil DT, Hofstetter WL, Kelsen DP, Blackstone EH (2017). Cancer of the esophagus and esophagogastric junction-major changes in the American joint committee on Cancer eighth edition cancer staging manual. CA Cancer J Clin.

[8] Japan Esophageal Society (2017). Japanese classification of esophageal Cancer, 11th edition: part I. Esophagus..

[9] Phillips AW, Lagarde SM, Navidi M, Disep B, Griffin SM (2017). Impact of extent of lymphadenectomy on survival, post neoadjuvant chemotherapy and transthoracic esophagectomy. Ann Surg.

[10] Udagawa H, Ueno M, Shinohara H, Haruta S, Kaida S, Nakagawa M, Tsurumaru M (2012). The importance of grouping of lymph node stations and rationale of three-field lymphoadenectomy for thoracic esophageal cancer. J Surg Oncol.

[11] Toh Y, Oki E, Minami K, Okamura T (2010). Follow–up and recurrence after a curative esophagectomy for patients with esophageal cancer: the first indicators for recurrence and their prognostic values. Esophagus..

[12] Miyata H, Yamasaki M, Kurokawa Y, Takiguchi S, Nakajima K, Fujiwara Y, Konishi K, Mori M, Doki Y (2011). Survival factors in patients with recurrence after curative resection of esophageal squamous cell carcinomas. Ann Surg Oncol.

[13] Abate E, DeMeester SR, Zehetner J, Oezcelik A, Ayazi S, Costales J (2010). Recurrence after esophagectomy for adenocarcinoma: defining optimal follow-up intervals and testing. J Am Coll Surg.

[14] Yamashita H, Nakagawa K, Tago M, Nakamura N, Shiraishi K, Ohtomo K (2005). Salvage radiotherapy for postoperative loco-regional recurrence of esophageal cancer. Dis Esophagus.

[15] Watanabe M, Mine S, Yamada K, Shigaki H, Baba Y, Yoshida N, Kajiyama K, Yamamoto N, Sano T, Baba H (2014). Outcomes of lymphadenectomy for lymph node recurrence after esophagectomy or definitive chemoradiotherapy for squamous cell carcinoma of the esophagus. Gen Thorac Cardiovasc Surg.

[16] Ichida H, Imamura H, Yoshimoto J, Sugo H, Kajiyama Y, Tsurumaru M, Suzuki K, Ishizaki Y, Kawasaki S (2013). Pattern of postoperative recurrence and hepatic and/or pulmonary resection for liver and/or lung metastases from esophageal carcinoma. World J Surg.

[17] Hiyoshi Y, Morita M, Kawano H, Otsu H, Ando K, Ito S, Miyamoto Y, Sakamoto Y, Saeki H, Oki E, Ikeda T, Baba H, Maehara Y (2015). Clinical significance of surgical resection for the recurrence of esophageal cancer after radical esophagectomy. Ann Surg Oncol.

[18] Nakajima Y, Kawada K, Tokairin Y, Tomita M, Miyake S, Kawano T (2016). Prognostic factors for post-recurrence survival in patients with thoracic esophageal squamous cell carcinoma after curative resection. Dig Surg.

[19] Depypere L, Lerut T, Moons J, Coosemans W, Decker G, Van Veer H (2017). Isolated local recurrence or solitary solid organ metastasis after esophagectomy for cancer is not the end of the road. Dis Esophagus.

[20] Aoyama J, Kawakubo H, Mayanagi S, Fukuda K, Irino T, Nakamura R, Wada N, Suzuki T, Kameyama K, Kitagawa Y (2019). Discrepancy between the clinical and final pathological findings of lymph node metastasis in superficial esophageal cancer. Ann Surg Oncol.

[21] Gottlieb-Vedi E, Kauppila JH, Malietzis G, Nilsson M, Markar SR, Lagergren J (2019). Long-term survival in esophageal cancer after minimally invasive compared to open esophagectomy: a systematic review and meta-analysis. Ann Surg.

[22] Takeno S, Takahashi Y, Moroga T, Kawahara K, Yamashita Y, Ohtaki M (2013). Retrospective study using the propensity score to clarify the oncologic feasibility of thoracoscopic esophagectomy in patients with esophageal cancer. World J Surg.

[23] Kauppila JH, Wahlin K, Lagergren P, Lagergren J (2019). Sex differences in the prognosis after surgery for esophageal squamous cell carcinoma and adenocarcinoma. Int J Cancer.

[24] Ando N, Kato H, Igaki H, Shinoda M, Ozawa S, Shimizu H, Nakamura T, Yabusaki H, Aoyama N, Kurita A, Ikeda K, Kanda T, Tsujinaka T, Nakamura K, Fukuda H (2012). A randomized trial comparing postoperative adjuvant chemotherapy with cisplatin and 5-fluorouracil versus preoperative chemotherapy for localized advanced squamous cell carcinoma of the thoracic esophagus (JCOG9907). Ann Surg Oncol.

[25] Shapiro J, van Lanschot JJ, Hulshof MC, van Hagen P, van Berge Henegouwen MI, Wijnhoven BP, van Laarhoven H, Nieuwenhuijzen GAP, Hospers GAP, Bonenkamp JJ, Cuesta MA, Blaisse RJB, Busch ORC, ten Kate F, Creemers GM, Punt CJA, Plukker JTM, Verheul HMW, Bilgen EJS, van Dekken H, van der Sangen M, Rozema T, Biermann K, Beukema JC, Piet AHM, van Rij C, Reinders JG, Tilanus HW, Steyerberg EW, van der Gaast A, CROSS study group (2015). Neoadjuvant chemoradiotherapy plus surgery versus surgery alone for oesophageal or junctional cancer (CROSS): long-term results of a randomised controlled trial. Lancet Oncol.

[26] Cunningham D, Allum WH, Stenning SP, Thompson JN, Van de Velde CJ, Nicolson M (2006). Perioperative chemotherapy versus surgery alone for resectable gastroesophageal cancer. N Engl J Med.

